# London and Liverpool Hospitals

**Published:** 1923-12

**Authors:** 


### LONDON AND LANCASHIRE HOSPITALS.

The following comparative figures relating to 113 London, 16 Liverpool hospitals, and the 17 hospitals of Manchester, have been compiled by Mr. F. G. D'Aeth, the Secretary of Liverpool Council of Voluntary Aid. The Manchester figures are based upon those contained in the annual report on the hospitals of that city, prepared by the City Treasurer, who is Hon. Sec. of the Manchester Voluntary Hospitals Committee. With regard to the London hospitals the income figure on which the percentages are based consists of all income received by the general funds of the various hospitals with the exception of money raised by the special appeal (total £243,115). The income figure for the Liverpool hospitals includes all money received during the yearb y the institutions (that is, general and other smaller funds). The total of the Voluntary Hospitals Commission grants (London £146,200, Manchester £12,485, and Liverpool £10,964) is included in the income, together with all legacies and donations for investment.

Comparative Table.

| | London. | Liverpool. | Manchester. |
|---|---|---|---|
| 1. *Annual Subscriptions* .. | Figure not available. | £20,867 | £55,469 |
| Percentage to Total Income .. .. | " | 7.0% | 20.3% |
| 2. *Hospital Saturday and Sunday Fund* .. | " | £17,150 | £14,517 |
| Percentage to Total Income .. .. | " | 5.8% | 5.8% |
| 3. *Income from Investments* | £505,549 | £37,301 | £51,476 |
| Percentage to Total Income .. .. | 19.8% | 12.7% | 18.8% |
| 4. *Patients' Payments* .. | £489,717 | £55,378 | £34,011 |
| Percentage to Total Income .. .. | 19.2% | 18.8% | 12.4% |
| 5. *Public Authorities and Approved Societies* .. | £255,446 | £33,773 | £38,997 |
| Percentage to Total Income .. .. | 10.4% | 11.4% | 14.2% |
| 6. *Earnings (Fees, etc.)* .. | £23,928 | £6,987 | £11,572 |
| Percentage to Total Income .. .. | 0.9% | 2.3% | 4.2% |
| 7. *Legacies* .. .. | £172,316 | £75,472 | £22,312 |
| Percentage to Total Income .. .. | 6.7% | 25.6% | 8.1% |

The percentages for the London hospitals are based on the figures given on pp. 30 and 31 of the King Edward's Fund Report.

